# Are narcissists more creative? Only if we believe it: How narcissism can relate to creativity

**DOI:** 10.3389/fpsyg.2022.1091770

**Published:** 2023-01-06

**Authors:** Yueting Ji, Haiyang Liu, Shengming Liu, Minya Xu, Zixiang Lin

**Affiliations:** ^1^Department of Organizational Behavior and Human Resource Management, Business School, Central University of Finance and Economics, Beijing, China; ^2^Division of Leadership, Management, and Organizations, Nanyang Business School, Nanyang Technological University, Singapore, Singapore; ^3^Department of Business Administration, School of Management, Fudan University, Shanghai, China; ^4^Department of Business Statistics and Econometrics, Guanghua School of Management, Peking University, Beijing, China; ^5^Policy Research Office, China National Petroleum Corporation, Beijing, China

**Keywords:** narcissism, creativity evaluation, personal reputation, political skill, impression managment

## Abstract

The relationship between narcissism and creativity has inspired interesting debates for decades. Drawing on a new perspective, the current study tried to explain how narcissism influences others’ creativity evaluation in the organizational context. Based on the theory of impression management, we suggested that narcissism and creativity may have a more complex relationship rather than a simple linear link. To test this relationship, we conducted a survey of 596 subordinates and 60 leaders in three high-technology companies. The result showed that there was an inverted U-shaped relationship between narcissism and creativity evaluation. Moreover, personal reputation mediated this curvilinear relationship and this relationship was significant only when narcissists were low in political skill. Theoretical and practical implications, limitations and future directions have also been discussed.

## Introduction

“We don’t settle for anything less than excellence.” -------Steve Jobs

Da Vinci, Nietzsche, Oscar Wilde, and Salvador Dali; such genii have the best minds in human history. They have left us with a fortune of thought, their famous stories illustrating the fine line between genius and insanity providing a source of great delight to explore. Their pomposity amazes the public, who laugh it off: How can we blame a person for simply being gifted? Similarly, in the organizational context, business leaders—such as Mark Zuckerberg, Elon Musk, and Donald Trump—are often self-aggrandizing and self-involved, eager to push their vision and products on the masses. It seems that there exists a common stereotype of highly creative or capable individuals as being narcissistic (Macdonald and Wilson, 2005; Smith and Webster, 2018). Anecdotes, stories, and biographies share the common view of the link between narcissists and creative talents.

The relationship between narcissism and creativity has likewise inspired interesting debates (Lebuda et al., 2021). Several scholars suggest no such relationship, in that narcissism may be an inevitable by-product of creative talent (Goncalo et al., 2010). Generally speaking, creative people spend a considerable deal of time alone, are often absorbed in their work to the point of obsession, and refuse to conform to social conventions; such behavior leads to the perception of narcissism to others. By contrast, given that narcissists are motivated to generate novel ideas to “stand out” and draw others’ attention, the opposite view is that narcissism may directly contribute to creativity (Raskin, 1980) and entrepreneurial behavior (Li et al., 2021). With personality traits involving grandiose self-views, sense of entitlements, and desire for uniqueness and superiority (Morf and Rhodewalt, 2001; Campbell et al., 2011), narcissists are compelled to engage in praiseworthy behaviors to affirm their favorable self-views (Resick et al., 2009). This view that narcissism is positively linked to creativity is gaining evidence in psychological fields, but has barely been tested in actual organizational contexts. Moreover, in literature, self-reported measures of creativity are often used (Raskin, 1980; Goncalo et al., 2010; Furnham et al., 2013), which may cause bias given that narcissists have a strong self-enhancement tendency and may view themselves more positively than others do (Grijalva and Harms, 2014). The difficulty to determine whether narcissists simply believe they are creative or actually demonstrate creative behaviors warrants investigation (Mao et al., 2021), therefore presenting considerable significance to examine the question: Is narcissism related to perceived creativity in real organizational contexts, and through which mechanism can narcissists achieve such evaluation?

The current study suggests that in actual organizations, rather than a simple linear link, narcissism and creativity may have a more complex relationship, such as a reversed U-shape mediated by personal reputation. On the basis of impression management theory, this study posits that narcissists are both motivated to manage their impression on others to achieve high creativity evaluation and unconsciously act to be perceived as creative individuals. Narcissists may intentionally gain admiration (motivation) while also (unconsciously) inspiring others to consider them to have leadership, confidence, and charm that contribute to their good reputation (Glad, 2002; Back et al., 2010), leading to high creativity evaluation. However, consistent with the Chinese philosophy of “Modest is the best,” when narcissism exceeds a moderate level, its dark side becomes so salient that others can view their antagonistic self-protection intentions as vulnerability, aggressiveness, arrogance, and grandiosity (Amabile, 1996; Back et al., 2010). These perceived impressions can be detrimental to the narcissist’s reputation, which in turn, influence others’ creativity evaluation. Thus, not all narcissists can win others’ good impressions; and only those with moderate narcissism level can achieve this feat.

In impression management field, a central topic that is gaining considerable attention is political skill, which is suggested to help various personalities achieve their social reputation. Hogan and Shelton (1998) argued that social skills serve as a moderator that can help or impede the interpretation of people’ motivations into observers’ evaluation. Accordingly, the present study posits that the relationship between narcissism and reputation is moderated by political skill, which refers to an interpersonal effectiveness construct that combines social understanding with the flexibility to adjust behaviors to fit the demands of the situation in ways that appear sincere, inspire trust and support, and effectively influence others (Ferris et al., 2005). Thus, individuals with high political skill can better manipulate others’ perceptions to underestimate their dark sides, thereby restricting the influence of narcissism. We propose that the inverted U-shape correlation of narcissism with both personal reputation and creativity may be significant only when narcissists have low political skill.

**Figure 1:**
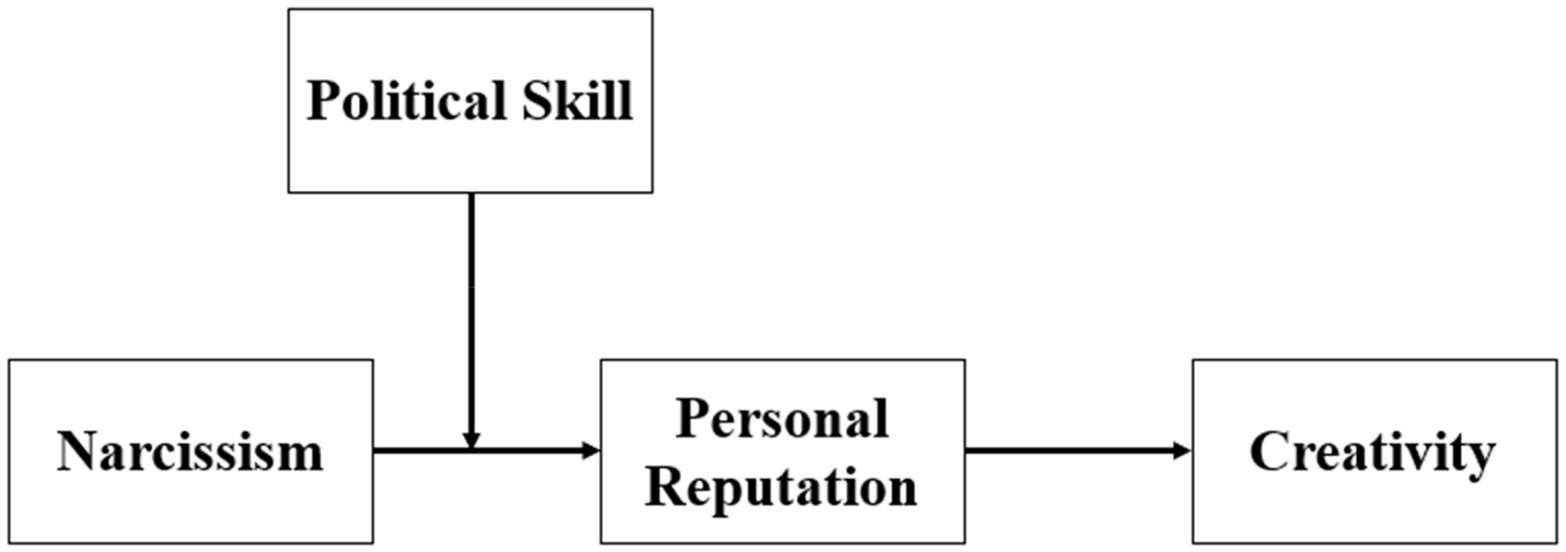
Conceptual model.

In sum, we examine the relationship between narcissism and creativity by building a moderated mediation model (Figure 1), which contributes to existing research in several ways. First, examining creativity as an outcome of narcissism enriches the present understanding by highlighting the potential “bright side” of a “dark trait,” given that narcissism is typically regarded as undesirable with severe implications for negative employee behavior (O’Boyle et al., 2012; Grijalva and Harms, 2014), such as counterproductive work behavior (Li et al., 2020). Second, this study contributes to narcissism literature by providing evidence demonstrating that moderate narcissism level has an optimal effect, complementing previous studies that simply suggest a positive relationship between narcissism and creativity (Furnham et al., 2013). Third, diverging from self-reports of creativity, we use other-rater (supervisor) reports of creativity in field study (Zhou et al., 2019), which can better explore the relationship between narcissism and true creativity. Finally, by exploring the mediating effect of personal reputation and moderating effect of political skill, we contribute to creativity research by examining and demonstrating the effect of personality on impression.

## Argument and hypothesis

### Narcissism and creativity: a new perspective of impression management

Greek Mythology tells the story of Narcissus, a proud young man who fell in love with his own reflection. Taken from his name, narcissism can be defined as a pervasive pattern of grandiosity, self-importance, and self-regard (Resick et al., 2009). Narcissists are often described as arrogant, confident, charming, always a leader, have a strong desire to be unique, need attention and admiration, and are likely to take risks (Bogart et al., 2004; Chatterjee and Hambrick, 2011). This trait involves both the bright side of self-enhancement and the dark side of self-protection, characteristics that lead to contrary outcomes in the organization (Küfner et al., 2013); thus, the effect of narcissism on processes and consequences have always been paradoxical. In terms of the bright side, narcissists are highly charming, self-assured, and more successful in short-term romantic relationships (Rhodewalt and Eddings, 2002; Holtzman and Strube, 2010) as well as humorous (Back et al., 2010). In terms of the dark side, narcissists may impede cooperation with others (Blair et al., 2008), have high correlations with aggressive behaviors (e.g., Bushman et al., 2003) and counterproductive work behaviors (Penney and Spector, 2002).

Most importantly, the relationship between narcissism and creativity currently attracts much academic attention (Elsbach and Kramer, 2003; Brunell et al., 2008; Goncalo et al., 2010; Mao et al., 2021). The possible reason may be the anecdotes, stories, and biographies that provide the impression that the most talented people are self-aggrandizing and narcissistic. Whether or not this logic is true has become the center of debate (John and Robins, 1994; Farwell and Wohlwend-Lloyd, 1998; Dugosh and Paulus, 2005). By using a sample of 1,375 young adults, Martinsen et al. (2019) suggested that narcissism can contribute to creativity. Yet, laboratory experiments such as by Farwell and Wohlwend-Lloyd (1998) revealed that narcissists are not necessarily more creative than their peers. In addition, narcissism is found to only cause a devious evaluation of creativity (Goncalo et al., 2010), which in turn is actually influenced by the traits of storytellers (Elsbach and Kramer, 2003).

Although the relationship between narcissism and creativity has undergone considerable exploration, several limitations still need further verification. First, existing research mainly used experimental method and may ignore complex real organizational contexts. Second, previous research mainly focuses on the motivations of narcissists regarding creativity, demonstrating the efforts they exert to gain others’ admiration or attention (Dugosh and Paulus, 2005). However, the social side of narcissists’ creativity—that is, how others’ perceptions or evaluations can influence such process—has been largely overlooked. Therefore, the present study focuses on others’ evaluation of narcissists’ creativity in the organizational context and proposes an inverted U-shape correlation. Narcissists’ tendencies toward impression management bring them better creativity evaluations. In other words, to explore the relation between narcissism and individual creativity, we proposed a new perspective that the evaluation of narcissists’ creativity can be a result of managed personal reputation.

### Narcissism and personal reputation

Basically, one’s personal reputation is a summary of the evaluation and perception of outside cues, including efforts and creativity (Hogan and Shelton, 1998). In other words, “personal reputation is the link between the actor’s efforts to achieve acceptance and status and how those efforts are evaluated by observers” (Hogan and Holland, 2003, p.100). Highhouse et al. (2009) stated that personal reputation is a shared impression; the amalgamation of individual impressions. This study posits that, to obtain good personal reputation, narcissists can use impression management, a ubiquitous act of both consciously and unconsciously controlling “the images that are projected in … social interactions” by different patterns of behaviors. With the use of impression management, individuals can achieve greater career success, obtain job offers (Ellis et al., 2002) and become highly evaluated to have good organizational citizenship (Bolino, 1999; Hui et al., 2000; Rioux and Penner, 2001).

We believe that narcissists are highly motivated to carry out impression management to gain a personal reputation. The reasons may be quite straightforward. First, as suggested by Wink (1991), narcissists have high vulnerability–sensitivity, which indicates that they are defensive, anxious, emotional, and discontented. On this basis, Atlas and Them (2008) suggested that narcissists are highly sensitive to negative feedback on their performance or appearance while Morf and Rhodewalt (2001) suggested that they can be deeply hurt by criticism. In addition, narcissists are believed to have grandiosity–exhibitionism, which implies their power orientation, manipulativeness, self-dramatization, and broad interests (Wink, 1991). As such, narcissists seriously consider projecting an image of competence (Elliot and Thrash, 2001), having a very strong need for others’ approval, attention, and recognition of their valuable contributions (John and Robins, 1994). Therefore, narcissists have considerable motivation to avoid negative feedback and seek positive evaluations, and are thus driven to manage their personal reputations. This argument has considerable evidence. For instance, Gardner and Pierce (2011) found that narcissists can be sufficiently skilled at consciously managing their impression on others. John and Robins (1994) also suggested that narcissists repetitively stress on their achievements and express their vision enthusiastically to achieve a good reputation.

Thus, narcissists—by definition, those who think highly of themselves—attempt to use every means and endeavors to maintain good personal reputations. More importantly, the desired admiration drives narcissists to actively display unique positive characteristics to maintain their personal reputation. However, at the same time, too much narcissism can also damage personal reputation. When the level of narcissism increases to a certain point, its relationship with reputation changes. Atlas and Them (2008) suggested that overly narcissistic individuals may evolve and become uncaring about others’ approval or aversion. When narcissists’ need for others’ approval, attention, and recognition decline, narcissists’ conscious impression management recedes accordingly. In addition, excessive narcissistic behaviors—such as being selfish, excessively dominant, aggressive, and arrogant (e.g., Colvin et al., 1995; Morf and Rhodewalt, 2001)—can be perceived by others as incompetent or annoying (Tsang et al., 2010). Therefore, personal reputation may deteriorate due to excessive narcissism.

Moreover, compared with their peers, narcissistic individuals have a significantly higher view of themselves. Park et al. (2013) found that narcissists’ self-ratings are excessively positive and much higher than those of their friends. This discrepancy and overconfidence may cause trouble. As overconfidence increases, their accuracy of problem-solving declines, which can lead to petty mistakes and defective work (Vallone et al., 1990). As narcissists become increasingly self-aggrandizing, their work quality may deteriorate, damaging their personal reputation. As such, we believe the relationship between narcissism and personal reputation is decided by the extent of the former. We propose the following hypothesis.

*Hypothesis 1*: Narcissism and personal reputation have an inverted U-shaped relationship.

### Narcissism, personal reputation, and creativity

The collection of perceptions of individuals from others represent personal reputation, which can be influenced by impression management and bring positive outcomes to individuals such as performance evaluations, promotions, and compensation (Ferris et al., 2003; Zinko et al., 2007; De Cremer and Sedikides, 2008). Personal reputation can also influence others’ evaluation of creativity of the focal individual. The reasons are three-fold. First, as Hogan and Shelton (1998) stated, built on individuals’ past behaviors as the best reference, personal reputation can serve as a valid assessment. Individuals with higher personal reputations are often perceived as more competent (Gioia and Sims, 1983), higher in status (Hochwarter et al., 2007), and more trustworthy (Ostrom, 2003) than their peers. To a certain extent, personal reputation is a signal of all these positive characteristics, serving as a reference when one needs to evaluate others’ creativity and especially when clear and exact information is difficult to acquire. In others’ eyes, a person with good personal reputation has a greater probability to be trustworthy and do better work (Ferris et al., 2003; Hochwarter et al., 2007; Zinko et al., 2007). Thus, one’s subjective creativity evaluation can be highly influenced by personal reputation.

Second, individuals with good personal reputations have greater autonomy than their peers, which indicates greater freedom to be creative (Humphrey et al., 2007) because good personal reputation can reduce ambiguity and the need for others’ monitoring (Zinko et al., 2007). Agency theory and current research provide evidence that individuals with high personal reputation are discretionary, because the qualities of their actions are predictable and thus, they do not need supervision (Hayward et al., 2004). Such autonomy supports individuals with more freedom and fewer constrictions, which facilitates emerging novel and creative actions.

Finally, personal reputation is also believed to bring about the halo effects that promote others’ evaluation toward the focal person in almost every aspect (Nisbett and Wilson, 1977). For one thing, good personal reputation positively relates to power (Gioia and Sims, 1983; Pfeffer, 1992; Zinko et al., 2007) and can carry out formal or informal authority that others may wish to identify. For another, personal reputation is a social factor associated with others’ evaluation such as perceived performance (Herbig and Milewicz, 1993; Ferris et al., 2003). Therefore, others have greater expectations and set them higher goals. Personal reputation has a positive relationship with perceived—regardless of actual—performance (Dossett and Greenberg, 1981; Herbig and Milewicz, 1993; Kierein and Gold, 2000), and thereby contributes to a positive effect on perceived usefulness and novelty when evaluating the individual’s creativity. Therefore, people tend to believe that those with fine personal reputations are capable of generating useful and novel ideas. Thus, we present the following hypothesis.

*Hypothesis 2*: Personal reputation is positively related to creativity evaluation.

On the basis of the above arguments, we propose that narcissists have inner motivations to consciously manage their impression on others and form their personal reputations, further influencing their creativity evaluation. However, narcissism that surpasses a certain level may give rise to its dark side—“too much confidence” and “care less about others’ opinion.” Revelation of this dark side of narcissism can damage the personal reputation, which also destruct the creativity evaluation. Therefore, on the whole, we propose that:

*Hypothesis 3*: Personal reputation mediates the inverted U-shaped relationship between narcissism and creativity.

### Political skill as an important boundary condition

Defined as “the ability to effectively understand others at work, and to use such knowledge to influence others, to act in ways that enhance one’s personal and/or organizational objectives” (Ahearn et al., 2004, p. 311), political skill is found to convey honest and believable messages (Riggio et al., 1987) and has a positive relationship with trust, job satisfaction, perceived organizational support, job tension, and workplace outcomes (Treadway et al., 2004; Ferris et al., 2005). Based on social influence theory (Harris et al., 2007), individual characteristics as political skill have an effective influence on impression management strategies and the ability to understand and manage dynamic relationships with targets is critical for success. Harris et al. (2007) demonstrated that apart from the main effect, political skill also serves as a moderator between impression management strategies and work outcomes.

Based on the previous study, we propose that political skill may also moderate the efficacy of narcissists’ impression management. Specifically, high level of political skill would reduce the effect of narcissism on personal reputation and creativity evaluation for the following reasons. Political skill enables individuals to use social cues to perceive and understand others’ feelings in different situations and accordingly adjust their behaviors to gain an advantageous influence (Treadway et al., 2007). Furthermore, individuals high in political skill can become aware and mask both their conscious and unconscious behaviors regardless of their positive or negative effects on others’ evaluations. As stated by Ferris et al. (2005, p. 128), “people high in political skill not only know precisely what to do in different social situations at work, but how to do it in a manner that disguises any ulterior, self-serving motives, and appears to be sincere.” Thus, for those with high political skill, instead of the personality, their interpersonal ability determine the outcome of impression management. By contrast, individuals with low political skill can hardly restrict the narcissism effect on others’ impressions and retain the previously discussed inverted U-shape relationship between narcissism, personal reputation, and creativity. Consequently, we propose the following hypothesis.

*Hypothesis 4*: Political skill moderates the relationship between narcissism and personal reputation: the relationship is inverted U-shaped only when individuals’ political skill is low.

*Hypothesis 5*: Political skill moderates the indirect effect of narcissism on creativity evaluation via personal reputation: the inverted U-shaped relationship is significant only when individuals’ political skill is low.

## Research context

### Sample and procedure

We conducted a two-wave, three-source survey study to test our hypotheses. Data are collected in one high-tech industrial development zone in Southern Mainland China for the survey. Three high-technology companies are approached to distribute questionnaires to group leaders and their team members on-site, and nearly 95 R & D teams carrying out creative tasks are selected as respondents. At time 1, questionnaires were distributed to 816 employees, who are asked to rate their own narcissism, political skill, and demographics. Respondents are also randomly asked to rate one of their colleagues’ personal reputations in the same team, such that each individual gained one peer rating. Subsequently, we retrieved 633 questionnaires. After 1 week, we ask the employees’ 60 leaders to rate their creativity evaluation, finding 596 that successfully match the followers’ questionnaires.

Among the employees, 332 (55.7%) were male, mean age was 31.71 years old (SD = 8.86). Employees’ average education was 14.79 years (SD = 5.48), average job tenure was 6.25 years (SD = 6.37), and average organization tenure was 4.64 years (SD = 5.36).

### Measures

#### Narcissism (Time 1)

Employee narcissism was measured by NPI-16 (Ames et al., 2006). The NPI-16 is a 16-item forced-choice questionnaire. Sample items are “I like to be the center of attention (1 = yes, 0 = no)” and “I think I am a special person (1 = yes, 0 = no).” The reliability coefficient for this scale was 0.70.

### Political skill (Time 1)

Employee’s political skill was measured by 18 items from the Political Skill Inventory developed by Ferris et al. (2005). This inventory includes four dimensions, such as networking ability, apparent sincerity, social astuteness and interpersonal influence. Sample item includes “I am good at using my connections and networks to make things happen at work (1 = totally disagree, 5 = totally agree).” The reliability coefficient for this scale was 0.90.

### Personal reputation (Time 1)

Employees’ personal reputation is reported by their peers. In each group, we randomly select one peer to rate the focal person’s personal reputation. We used the 12 items developed by Hochwarter et al. (2007). We adjust their reference from “I” to “He or she,” sample items include “He or she is regarded highly by others” and “If people want things done right, they ask him or her to do it (1 = totally disagree, 5 = totally agree).” In addition, we add one overall judgment item into our study, this item refers to “Generally speaking, he or she has a good personal reputation.” The reliability coefficient for 13 items was 0.91.

### Creativity evaluation (Time 2)

Employees’ creative evaluation was rated by their leaders using the six-item scale from Madjar et al. (2011). Sample items include “This follower is a good source of highly creative ideas” and “Uses previously existing ideas or work in an appropriate new way” (1 = totally disagree, 5 = totally agree). The reliability coefficient for this scale was 0.83.

### Control variables

We controlled for several variables, including employees’ gender (1 = male, 0 = female), age, education years, organization tenure, and job tenure, because these demographics have been found to influence supervisor-rated employee creativity (Ng and Feldman, 2013; Liu et al., 2016).

## Analytical strategies

To segment the variance from different organizational levels (team members and team levels) in hypothesis testing, we conducted hierarchical linear models (HLM) with Stata 12.1 (Rabe-Hesketh and Skrondal, 2008) to test our hypotheses. Even though our hypotheses did not contain level 2 variables, it has been suggested that we should control the multilevel effects because employees are nested in groups in our sample (Edwards and Lambert, 2007). We follow the suggestion by Rabe-Hesketh and Skrondal (2008) to control these effects. To have a computational advantage by reducing nonessential multicollinearity between the linear terms and their quadratic counterparts (Aiken et al., 1991), we treat the independence variable and moderate variable in models with a grand-mean-centered process (Hofmann and Gavin, 1998). The following HLM equations were established in our analysis:

*Y=*$b_{c}C$*+*$b_{1}$*X+*$b_{2}$*Z++*$b_{3}X^{2}$*+e* (1)

*Y=*$b_{c}C$*+*$b_{1}$*X+*$b_{2}$*Z+*$b_{3}X^{2}$*+*$b_{4}$*XZ +*$b_{5}X^{2}$*Z + e* (2)

*M=*$b_{c}C$*+*$b_{1}$*X+*$b_{2}$*Z+*$b_{3}X^{2}$*+*$b_{4}$*XZ +*$b_{5}X^{2}$*Z + e* (3)

*Y=*$b_{c}C$*+*$b_{1}$*X+*$b_{2}$*Z +*$b_{3}X^{2}$*+*$b_{4}$*XZ +*$b_{5}X^{2}$*Z + M + e* (4)

where *Y* was creative evaluation, *C* was the vector of control variables, *X* was narcissism, *M* was reputation and *Z* was political skill. We entered the variables into the regression equation in five steps. First, control variables (*C*) were entered along in the equation in step 1. Second, the main effect of narcissism (*X*) and political skill (*Z*) were entered in step 2. Third, to test the quadratic relationship in Hypothesis 1 we added the squared term (*X*^2^) in step3. Next, the interaction term of both linear interaction term (*XZ*) and quadratic interaction term (*X*^2^*Z*) were added to demonstrate the moderate effect in Hypothesis 4. In addition, to test the moderated mediation model (involving Hypotheses 2, 3, and 4), we followed the analysis approach suggested by Edwards and Lambert (2007). They suggested a general analytical framework that integrates moderated regression analysis and path analysis, which clarifies how moderator variables influence the paths that constitute the direct, indirect, and total effects of mediated models. In addition, to better analyze the conditional indirect effect size and confidence interval, we followed the Monte Carlo method suggested by Bauer et al. (2006) to test the multilevel conditional indirect confidence interval.

## Results

### Confirmatory factor analysis

To verify the variables measured in our research captured separate constructs, we conducted confirmatory factor analyses. Following recommendations by Little et al. (2002), we built parcels for scales with more than five items using the items-to-construct balance technique. Results showed that the four-factor model (e.g., narcissism, political skill, personal reputation, and creativity evaluation) not only fit the data fairly (*χ*^2^ [21] = 123.29, *p* < 0.01; CFI = 0.95, TLI = 0.92, SRMR = 0.03, RMSEA = 0.09), but was also better than a few alternative models, including the three-factor model with narcissism and political skill combined (*χ*^2^ [24] = 309.14, *p* < 0.01; CFI = 0.87, TLI = 0.81, SRMR = 0.08, RMSEA = 0.14; Δ*χ*^2^ [3] =185.85, *p* < 0.01), the three-factor model with narcissism and personal reputation combined (*χ*^2^ [24] = 325.58, *p* < 0.01; CFI = 0.86, TLI = 0.79, SRMR = 0.08, RMSEA = 0.15; Δ*χ*^2^ [3] = 202.29, *p* < 0.01), and the three-factor model with political skill and personal reputation combined (*χ*^2^ [24] = 570.15, *p* < 0.01; CFI = 0.75, TLI = 0.63, SRMR = 0.08, RMSEA = 0.20; Δ*χ*^2^ [3] = 446.86, *p* < 0.01). thereby providing support for the construct validity.

### Hypothesis testing

Table 1 shows the means, standard deviations, and correlations among the studied variables of current study. Employee narcissism was significantly correlated with creativity evaluation (*r* = 0.21, *p* < 0.01). In addition, both political skill (*r* = 0.26, p < 0.01) and reputation (*r* = 0.45, *p* < 0.01) were significantly correlated with creativity.

**Table 1:** Descriptive statistics, reliability coefficients, and correlations.

	Variable	*M*	SD	1	2	3	4	5	6	7	8	9
1	Age	31.71	8.86									
2	Gender	0.44	0.50	−0.08**								
3	Education	14.79	5.48	−0.02	−0.03							
4	Org tenure	4.64	5.36	0.54**	−0.05	−0.19**						
5	Job tenure	6.25	6.37	0.64**	−0.01	−0.14**	0.75**					
6	Narcissism	0.29	0.18	−0.04	0.00	−0.17**	0.02	−0.05	**0.72**			
7	Political Skill	3.53	0.47	0.12**	−0.05	0.01	0.09*	0.07	0.23**	**0.90**		
8	Personal Reputation	3.49	0.43	−0.01	−0.01	0.01	0.13**	0.11**	0.17**	0.46**	**0.91**	
9	Creativity evaluation	3.36	0.44	0.07	−0.02	0.02	0.16**	0.15**	0.21**	0.47**	0.65**	**0.82**

*N* = 596; Cronbach’s alphas appear on the diagonal on bold. ^*^p < 0.05, ^**^p < 0.01.

**Figure 2:**
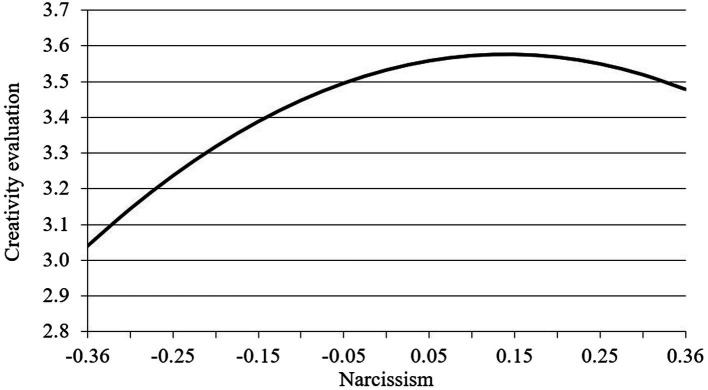
The relationship between narcissism and creativity evaluation.

Hypothesis 1 states that there is an inverted U-shaped relationship between narcissism and creativity evaluation. As shown in Table 2, after entering the squared term of narcissism, the narcissism squared reached significance on creativity evaluation (*b*_3_ = −0.84, *p* < 0.01, model 5) which means there is a curvilinear relationship between narcissism and creativity evaluation. Thus, Hypothesis 1 was supported. Finger 2 depicts this relationship between employee narcissism and creativity evaluation of his/her leader. As we can see, after the middle level of narcissism, the employee’s creativity evaluation begins to decrease. Before the culmination, the relationship between narcissism and one’s creativity evaluation was positive (Figures 2).

**Table 2:** Results of hierarchical linear modeling (HLM).

Predictors	Reputation			Creativity evaluation				
	Model 1	Model 2	Model 3	Model 4	Model 5	Model 6	Model 7	Model 8
Intercept	3.57**	3.58**	3.54**	3.44**	3.43**	3.33**	3.43**	3.34**
Controls								
Age ($b_{c1}$)	−0.01	−0.00	−0.01*	−0.01*	−0.01*	−0.01	−0.01*	−0.00
Gender ($b_{c2}$)	0.01	0.01	−0.00	−0.01	−0.00	−0.00	−0.01	−0.01
Education ($b_{c3}$)	0.00	0.00	0.01	0.00	0.00	0.00	0.01	0.00
Org tenure ($b_{c4}$)	0.00	0.00	0.00	0.01	0.01	0.00	0.01	0.00
Job tenure ($b_{c5}$)	0.01*	0.01*	0.01*	0.01**	0.01**	0.01	0.01**	0.01
Main studies variables								
Narcissism ($b_{1}$)	0.09	0.25*	0.20	0.21*	0.35**	0.20*	0.30**	0.18
Political skill ($b_{2}$)	0.40**	0.39**	0.29**	0.41**	0.41**	0.19**	0.31**	0.14**
Narcissism squared ($b_{3}$)		−0.96**	−1.44**		−0.84*	−0.32	−1.13**	−0.42
Interactions								
Narcissism × political skill ($b_{4}$)			−0.10				−0.35	−0.31
Narcissism squared × political skill ($b_{5}$)			2.91**				2.51**	0.99
Personal reputation ($b_{6}$)						0.55**		0.54**
Personal reputation × political skill ($b_{7}$)								0.09
Pseudo *R* square	0.02	0.43	0.55	0.27	0.40	0.50	0.54	0.63

*N* = 596. ^*^p < 0.05, ^**^p < 0.01.

Hypothesis 2 suggests that the employee’s reputation is positively related to creativity evaluation. As shown in Table 1, reputation (*r* = 0.45, *p* < 0.01) was significantly correlated with creativity evaluation. In addition, Model 6 in Table 2 also demonstrates that reputation is significantly related to one’s creativity evaluation (*b*_6_ = 0.55, *p* < 0.01). Therefore, Hypothesis 2 was supported. Hypothesis 3 involves the mediating effects of reputation on the quadratic relationship between narcissism and creativity evaluation. To demonstrate such hypothesis, two steps were conducted. First, as shown in Table 2, after entering the squared term of narcissism in Model 2 the narcissism squared reached significance on reputation (*b*_3_ = −0.96, *p* < 0.01). Then, we want to see whether the quadratic effects of narcissism on creativity evaluation still exist after controlling reputation. Model 5 suggests that without controlling reputation, the coefficient of narcissism quadratic term is significant (*b*_3_ = −0.84, *p* < 0.01). However, Model 6 demonstrates that the quadratic term of narcissism is no longer significant when reputation entered the equation (*b*_3_ = −0.32, ns). Then Hypothesis 3 was demonstrated.

**Figure 3:**
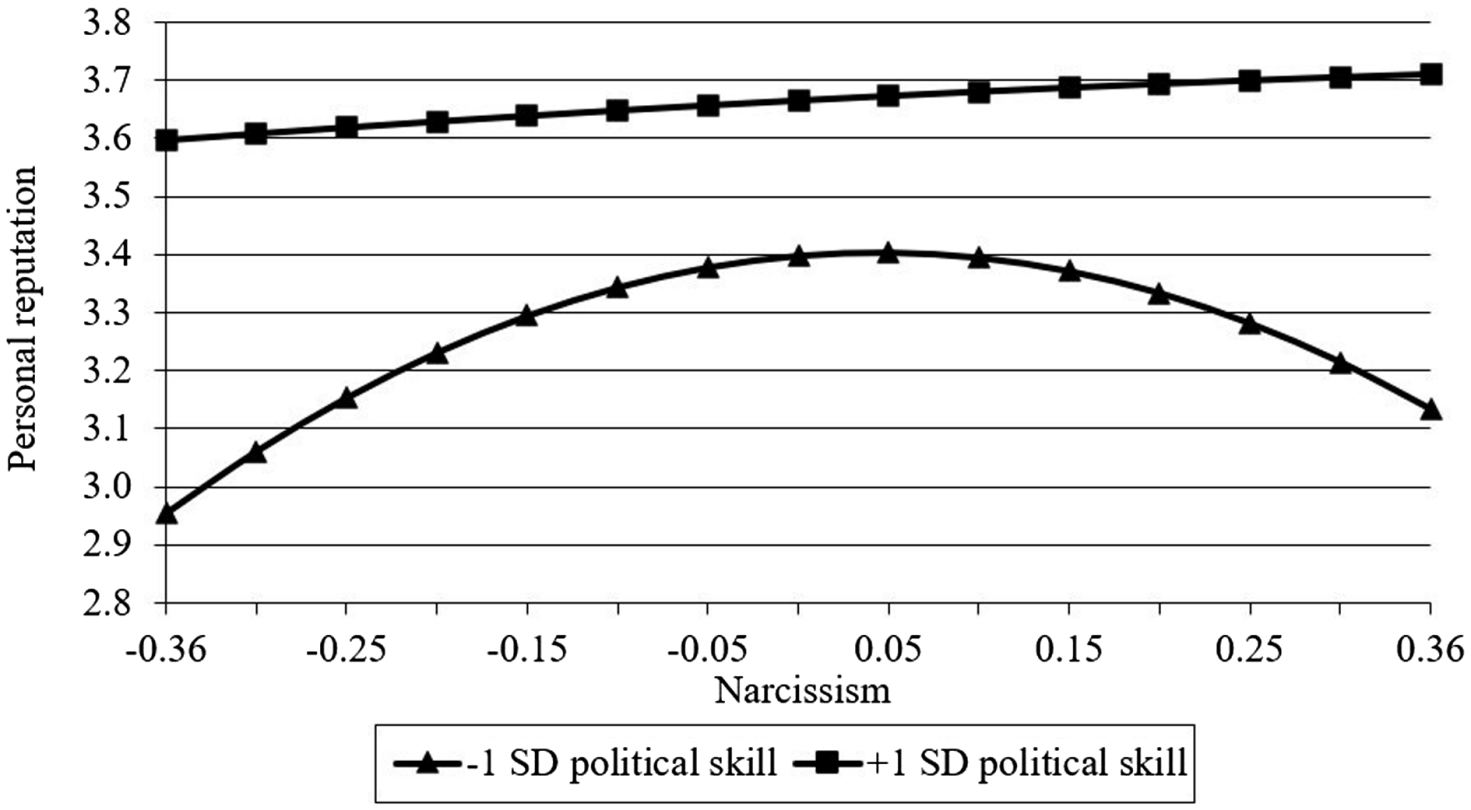
The moderating effect of political skill on the relationship between narcissism and personal reputation.

**Figure 4:**
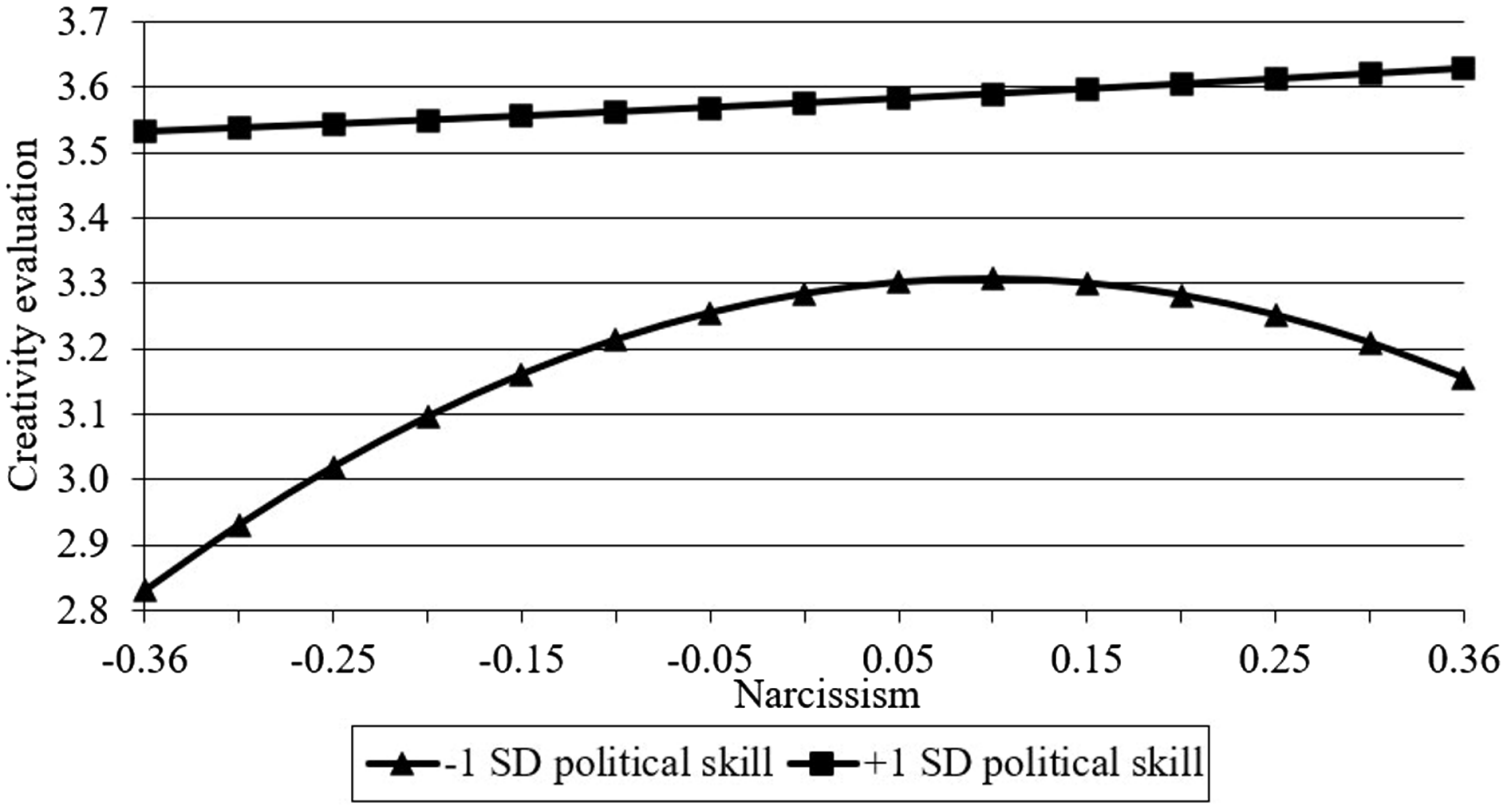
The moderating effect of political skill on the relationship between narcissism and creativity evaluation.

Hypotheses 4 and 5 involve a moderated mediated model. To test such model, we conduct several steps to demonstrate it suggested by Edwards and Lambert (2007). First, we demonstrate that political skill moderates the effect of narcissism on both reputation and creativity evaluation. Results in Table 2 indicated that although the interaction of narcissism and political skill was not significant for reputation (*b*_4_ = −0.10, ns, model 3) and creativity evaluation (*b*_4_ = −0.26, ns, model 7), the interaction of squared narcissism and political skill reached significance for reputation (*b*_5_ = 2.91, *p* < 0.01, model 3) and creativity evaluation (*b*_5_ = 2.52, *p* < 0.01, model 7). Further, we take a simple slope analysis (Aiken et al., 1991) to probe the moderating effect of political skill. When the political skill is high (1 SD above the mean), neither narcissism nor squared narcissism was significant for reputation (narcissism: *b* = 0.16, ns; squared narcissism: *b* = −0.09, ns) and creativity (narcissism: *b* = 0.13, ns; squared narcissism: *b* = 0.04, ns) which means that narcissism does not have a significant effect on dependence variables. In contrast, when the political skill is low (1 SD below the mean), only squared narcissism has a significant effect on reputation (narcissism: *b* = 0.25, ns; squared narcissism: *b* = −2.80, *p* < 0.01) and creativity (narcissism: *b* = 0.46, *p* < 0.01; squared narcissism: *b* = −2.30, *p* < 0.01) indicates that the relationship between narcissism and dependent variables showed a positive trend at lower levels of narcissism and a negative trend at higher levels of narcissism. In addition, at any point of narcissism, the reputation or creativity at high-level political skill is higher than low-level political skill. Hence, the moderating effects of political skill on the relationship between quadratic terms of narcissism on both reputation and creativity evaluation have been demonstrated. Figures 3 and 4 describe the relationship between narcissism and both reputation and creativity at two levels of political skill which are plus and minus one standard deviation, respectively.

In the second step, we want to demonstrate that political skill can moderate the indirect effects of the quadratic term of narcissism on creativity evaluation through reputation. We conducted the moderated mediation hypotheses tests using the analysis approach suggested by Edwards and Lambert (2007). In addition, we use the Monte Carlo method (Bauer et al., 2006) to estimate the conditional indirect effect confidence interval. The method suggests that when the 95% bias-corrected confidence interval (CI) of the indirect effect (a*b) excludes zero, this denotes a statistically significant indirect effect. When political skill is low (1 SD below the mean), the indirect effect of quadratic narcissism term on creativity evaluation through reputation is significant (a*b = −0.45, CI = [−0.83, −0.07]). In contrast, the indirect effect through reputation is not significant (a*b = 0.02, CI = [−0.40, 0.44]) when political skill is high (1 SD above the mean). Thus, Hypotheses 4 and 5 are supported.

## Discussion

The present study explains how narcissism influences others’ evaluations about their creativity from an impression management perspective in the organizational context. First, individuals with a moderate narcissism level are found to have a higher creativity evaluation than those with higher or lower narcissism levels. Second, others’ reported reputations mediate the relationship between narcissism and creativity. Third, narcissism influences creativity only when individuals have low political skill.

### Theoretical implications

The findings present several important theoretical implications. First, this study contributes to work on the relationship between narcissism and creativity by conceptualizing and examining whether this theory can complement real organizational contexts. The effects of narcissism on creativity have been established *via* substantial experiments (Goncalo et al., 2010), but do not adequately reflect the complex real-world contexts. Thus, conclusions about the relationship between narcissism and creative performance may be potentially inaccurate. Chinese philosophy “ZhongYong” also suggests that excessive positives may become negatives, and only moderate “positives” may achieve the best. The current study identifies a similar boundary condition of the proposed relationship, unveiling the mystery of the complicated effects of narcissism to a greater extent and showing its inverted and curvilinear U-shape.

Second, this study also contributes to creativity literature by explicitly extending the relationship between narcissistic personality, personal reputation, and creativity. Compared with the plethora of research on personality and creativity (for a review, see Feist, 1998), this study examines the potential mechanism that a particular personality can lead to a better personal reputation and eventually influence creativity evaluation from others. Thus, we enrich our understanding of how personality can be translated into creativity in organizational settings based on an impression management perspective. We also advance our understanding of the social process of how personality can influence creativity in organizations and reveal the importance of personal reputation (Liu et al., 2016).

Third, this study contributes to narcissism literature. In psychology, narcissism is one of the most enigmatic constructs because of its contradictory processes and consequence: the confidence and charisma of narcissists can bring tremendous energy to fascinate others, whereas their aggressiveness and lack of empathy can cause conflicts (Back et al., 2010). The effect of narcissism on performance and behavior also shows mixed results (Judge et al., 2006; O’Boyle et al., 2012). Our empirical findings promote narcissism research by providing evidence that its bright side is a dominant factor to improve personal reputation, but after exceeding a threshold, the dark side of narcissism becomes dominant and reduces personal reputation. Thus, narcissism has an optimum point that shows the best effect.

Finally, this study contributes to the theory and examination of impression management. Based on fundamental assumptions that such perspective mainly focuses on performance and career success, we hypothesize and test its applicability in creativity research by illustrating that narcissists use both conscious and unconscious methods to manage their impression on others, which in turn influences their reputation and creativity. This extension can shed light on future research on how creativity unfolds socially in organizations. Specifically, as an important impression management skill, political skill is proven to have a moderating role in the relationship between narcissism and creativity.

### Practical implications

Apart from theoretical contributions, this study also presents significant practical meaning to organizations. Narcissistic employees must be cautious about their double-edged personality while decision-makers must carefully consider their subjective evaluations. In the contemporary changing world, organizations need creative employees and leaders to survive, compete, and develop. In addition to the causes of creativity, its methods of evaluation also need research attention. However, creativity is often evaluated subjectively rather than by objective criteria (Sutton and Hargadon, 1996; Goncalo et al., 2010). This study reveals that, interestingly, personality traits such as narcissism can influence others’ evaluations about people. Thus, managers that need to select and adopt creative ideas without an ideal object criterion must remain vigilant on whether their decisions are contaminated by the targets’ reputations and personalities. Therefore, an objective evaluation system must be established. In addition, narcissists must also realize that such personality trait is a double-edged sword, and therefore need to control its damage to themselves and others. One possible way is to improve their political skill to better adjust their personality effect and obtain a more stable and higher reputation.

### Future directions and limitations

Although this study presents the above strengths, several limitations must be noted when interpreting the findings. First, as a cross-sectional study, the causal inference is limited. Nevertheless, adopting multiple, diverse methodologies can provide certain advantages and disadvantages (Wiesenfeld et al., 2007). Future studies can adopt a longitudinal design to verify our findings.

Second, creativity is a complex construct with many potential ways of measurement; thus, this study only demonstrates a definitive link between narcissism and one way of measured creativity. Therefore, future studies can use different measuring of creativity, objective and subjective, and see whether narcissism has consistent effects.

Third, narcissism is not the only personality that influences creativity and impression management. Future research can examine other traits that can increase people’s skills in impression management, such as positive self-evaluation, self-acceptance confidence, self-esteem, or self-efficacy (Tierney and Farmer, 2004). For example, individuals with high self-monitoring may convince others to believe in their creativity by detecting different evaluation cues of creativity in different contexts (Snyder, 1974).

## Data availability statement

The raw data supporting the conclusions of this article will be made available by the authors, without undue reservation.

## Author contributions

All authors listed have made a substantial, direct, and intellectual contribution to the work and approved it for publication.

## Funding

Haiyang Liu’s work on this research was supported by the Nanyang Technological University, Singapore, under its Start-Up-Grant (SUG #022274-00001). Shengming Liu’s work on this research was supported by the National Natural Science Foundation of China (72002038).

## Conflict of interest

ZL is employed by China National Petroleum Corporation.

The remaining authors declare that the research was conducted in the absence of any commercial or financial relationships that could be construed as a potential conflict of interest.

## Publisher’s note

All claims expressed in this article are solely those of the authors and do not necessarily represent those of their affiliated organizations, or those of the publisher, the editors and the reviewers. Any product that may be evaluated in this article, or claim that may be made by its manufacturer, is not guaranteed or endorsed by the publisher.

## Supplementary material

The Supplementary material for this article can be found online at: https://www.frontiersin.org/articles/10.3389/fpsyg.2022.1091770/full#supplementary-material
