# Supplementary material for: Are narcissists more creative? Only if we believe it: How narcissism can relate to creativity
Source: Front Psychol. 2023 Jan 6;13:1091770. doi: 10.3389/fpsyg.2022.1091770 (PMC9853005; doi:10.3389/fpsyg.2022.1091770)
Supplement: Supplementary file 1 [file Data_Sheet_1.docx]

**Appendix A: Measures Detail**

**Narcissism**

16-item pair measure of narcissism (Ames, Rose, and Anderson, 2006):

| Narcissistic response | Non-narcissistic response |
| --- | --- |
| I know that I am good because everybody keeps telling me so | When people compliment me I sometimes get embarrassed |
| I like to be the center of attention | I prefer to blend in with the crowd |
| I think I am a special person | I am no better or nor worse than most people |
| I like having authority over people | I don‘t mind following orders |
| I find it easy to manipulate people | I don’t like it when I find myself  manipulating people |
| I insist upon getting the respect that is due me | I usually get the respect that I deserve |
| I am apt to show off if I get the chance | I try not to be a show off |
| I always know what I am doing | Sometimes I am not sure of what I am doing |
| Everybody likes to hear my stories | Sometimes I tell good stories |
| I expect a great deal from other people | I like to do things for other people |
| I really like to be the center of attention | It makes me uncomfortable to be the center of attention |
| People always seem to recognize my authority | Being an authority doesn’t mean  that much to me |
| I am going to be a great person | I hope I am going to be successful |
| I can make anybody believe anything I want them to | People sometimes believe what I tell them |
| I am more capable than other people | There is a lot that I can learn from other people |
| I am an extraordinary person | I am much like everybody else |

**Political skill**

The Political Skill Inventory (Ferris et al., 2005):

1. I spend a lot of time and effort at work networking with others.

2. At work, I know a lot of important people and am well connected.

3. I am good at using my connections and network to make things happen at work.

4. I have developed a large network of colleagues and associates at work who I can call on for support when I really need to get things done.

5. I spend a lot of time at work developing connections with others.

6. I am good at building relationships with influential people at work.

7. It is important that people believe I am sincere in what I say and do.

8. When communicating with others, I try to be genuine in what I say and do.

9. I try to show a genuine interest in other people.

10. I always seem to instinctively know the right things to say or do to influence others.

11. I have good intuition or savvy about how to present myself to others.

12. I am particularly good at sensing the motivations and hidden agendas of others.

13. I pay close attention to people’s facial expressions.

14. I understand people very well.

15. It is easy for me to develop good rapport with most people.

16. I am able to make most people feel comfortable and at ease around me.

17. I am able to communicate easily and effectively with others.

18. I am good at getting people to like me.

**Personal reputation**

Hochwarter et al. (2007) Reputation Scale:

1. This individual is regarded highly by others.
2. This individual has a good reputation.
3. This individual has the respect of his/her colleagues and associates.
4. This individual has the trust of his/her colleagues.
5. This individual is seen as a person of high integrity.
6. This individual is regarded as someone who gets things done.
7. This individual has a reputation for producing results.
8. People expect this individual to consistently demonstrate the highest performance.
9. People know this individual will produce only high-quality results.
10. People count on this individual to consistently produce the highest quality performance.
11. This individual has the reputation of producing the highest quality performance.
12. If people want things done right, they ask this individual to do it.

**Creativity evaluation**

Madjar et al. (2011) creative performance measures:

1. Is a good source of highly creative ideas.
2. Demonstrates originality in his/her work.
3. Suggests radically new ways for doing advertising.
4. Uses previously existing ideas or work in an appropriate new way.
5. Is very good at adapting already existing ideas or ads.
6. Easily modifies previously existing work processes to suit current needs.
